# Pseudoautosomal Region 1 Length Polymorphism in the Human Population

**DOI:** 10.1371/journal.pgen.1004578

**Published:** 2014-11-06

**Authors:** Martin A. Mensah, Matthew S. Hestand, Maarten H. D. Larmuseau, Mala Isrie, Nancy Vanderheyden, Matthias Declercq, Erika L. Souche, Jeroen Van Houdt, Radka Stoeva, Hilde Van Esch, Koen Devriendt, Thierry Voet, Ronny Decorte, Peter N. Robinson, Joris R. Vermeesch

**Affiliations:** 1 KU Leuven, Department of Human Genetics, Leuven, Belgium; 2 Institut für Medizinische Genetik und Humangenetik, Charité - Universitätsmedizin Berlin, Berlin, Germany; 3 UZ Leuven, Laboratory of Forensic Genetics and Molecular Archaeology, Leuven, Belgium; 4 KU Leuven, Department of Imaging & Pathology, Biomedical Forensic Sciences, Leuven, Belgium; 5 KU Leuven, Department of Biology, Laboratory of Biodiversity and Evolutionary Genomics, Leuven, Belgium; University of Wisconsin-Madison, United States of America

## Abstract

The human sex chromosomes differ in sequence, except for the pseudoautosomal regions (PAR) at the terminus of the short and the long arms, denoted as PAR1 and PAR2. The boundary between PAR1 and the unique X and Y sequences was established during the divergence of the great apes. During a copy number variation screen, we noted a paternally inherited chromosome X duplication in 15 independent families. Subsequent genomic analysis demonstrated that an insertional translocation of X chromosomal sequence into theMa Y chromosome generates an extended PAR. The insertion is generated by non-allelic homologous recombination between a 548 bp LTR6B repeat within the Y chromosome PAR1 and a second LTR6B repeat located 105 kb from the PAR boundary on the X chromosome. The identification of the reciprocal deletion on the X chromosome in one family and the occurrence of the variant in different chromosome Y haplogroups demonstrate this is a recurrent genomic rearrangement in the human population. This finding represents a novel mechanism shaping sex chromosomal evolution.

## Author Summary

The human sex chromosomes differ in sequence, except for homologous sequences at both ends, termed the pseudoautosomal regions (PAR1 and PAR2). PAR enables the pairing of chromosomes Y and X during meiosis. The PARs are located at the termini of respectively the short and long arms of chromosomes X and Y. The observation of gradual shortening of the Y chromosome over evolutionary time has led to speculations that the Y chromosome is “doomed to extinction.” However, the Y chromosome has been shaped over evolution not only by the loss of genes, but also by addition of genes as a result of interchromosomal exchanges. In this work, we identified males with a duplication on chromosome Xp22.33 of about 136 kb as an incidental finding during a copy number variation screen. We demonstrate that the duplicon is an insertional translocation due to non-allelic homologous recombination from the X to the Y chromosome that is flanked by a long terminal repeat (LTR6B). We show this translocation event has occurred independently multiple times and that the duplicated region recombines with the X chromosome. Therefore, the duplicated region represents an extension of the pseudoautosomal region, representing a novel mechanism shaping sex chromosomal evolution in humans.

## Introduction

The human sex chromosomes originate from an ancestral homologous chromosome pair. During mammalian evolution, these chromosomes lost homology due to progressive degradation of the Y chromosome. The decay of the Y chromosome started with the introduction of sex determination factors, which initiated subsequent cycles of suppressed recombination [1]. Two main mechanisms are usually invoked to explain the reduction of XY homology. Reduced recombination rates near the pseudoautosomal boundary (PAB) would result in an accumulation of mutations which ultimately result in the inability to recombine [2]. Suppressed recombination led to the gradual decline in recombination. In addition, a stepwise reduction of recombination has been observed in the mammalian Y chromosome. Based on the nucleotide divergence between the human X and Y chromosome, nine different regions, termed strata, can be distinguished [3], [4]. It has been speculated that chromosomal rearrangements, such as inversions, might explain the stepwise decrease in sequence similarity between genes ordered on the human X chromosome and their homologs (called “gametologs”) on the Y chromosome [3]. Nevertheless, comparative genomic analysis has failed to identify such inversions [5], [6]. Hence, the forces driving recombination suppression remain to be established.

The observation of a gradual demise of the Y chromosome has lead to speculations that, from an evolutionary perspective, the Y chromosome is doomed to extinction [7], [8]. In contrast, recent evidence suggests that gene loss has been limited over the past 25 million years [9]. However, the Y chromosome is not only shaped by loss of genes and gene functions, but also by addition of genes as a result of interchromosomal exchanges. An autosome to gonosome translocation occurred after the divergence of the placental mammals from marsupials, increasing the size of the eutherian gonosomes [10]. Similarly, chromosomal regions known to be autosomal in different mammals seem to have translocated to the subtelomeric region of the long arm of the X chromosome during great ape evolution and subsequently to both the X and Y chromosome subtelomeres during hominid evolution [11], [12]. There remain two regions of homology: the 2.7 Mb pseudoautosomal region 1 (PAR1) at the telomeres of the short arms and the 0.3 Mb human specific PAR2 at the termini of the long arms [2], [13]. These XY homologous regions are required for pairing and synapse formation resulting in the obligate cross-over which is required for proper chromosome segregation during mammalian meiosis. Although often perceived as stable, it is well known that genes in the human PAR1 show elevated divergence with their primate orthologs and high levels of structural polymorphism. [14]–[17].

Relative to other mammalian species with a characterized PAR, the human PAB (i.e. PAB1; the human specific PAR2 has no counterpart in other genomes) is positioned distally. The PAB maps within the gene coding for one of the XG blood group antigens [18], [19]. *XG* is disrupted on the Y chromosome, and thus lacks nine exons on its 3′ end. The PAB was probably created by the intrachromosomal transposition of a chromosome fragment including the sex-determining region Y (*SRY*) gene [20]. Over time the PAB has shifted about 240 bp into the PAR by attrition, accounting for the fact that the PAR is flanked by a 240 bp segment of reduced homology (∼77%) on its proximal side [21]. An Alu element is located at the human PAB, but this is believed to have inserted after the divergence of old world monkeys and great apes and therefore did not create the PAB [22]. Hence, the PAB has remained stable since the divergence of the great apes and is considered stable in the Catarrhini lineage [1], [22]. Here we demonstrate that a previously undiscovered PAR1 length polymorphism exists in the human population as a result of recent recurrent chromosomal rearrangements that shifts the PAB by 110 kb towards the centromere.

## Results

### Segmental X Chromosomal Duplication Inserted in Y

**Figure 1:**
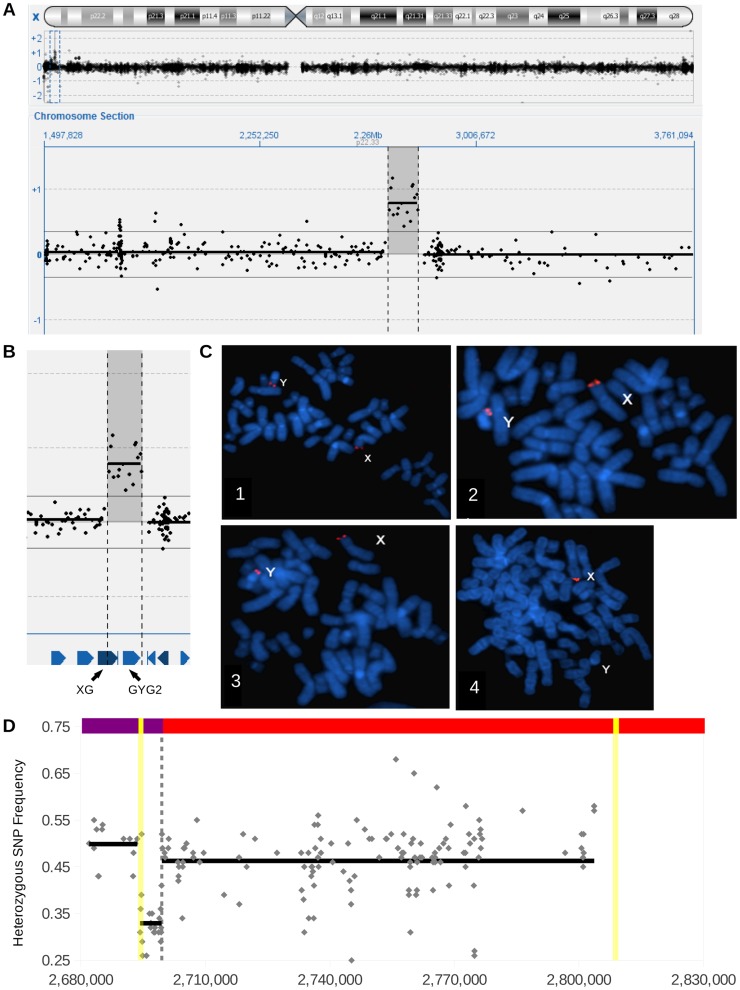
Identification of an X specific insertional translocation in Y. (A) Determining a duplication by array-CGH using a 180K Custom Microarray. The upper track shows an overview of the log2-ratio of the fluorescent signal across the entire X chromosome. The central track shows a zoomed in portion of the X chromosome, with the log2-ratio of the fluorescent signals on the Y Axis. (B) Further zooming in on the duplication, including the location of genes *XG* and *GYG2*. (C) FISH results for a father carrier (1, 3) and male control (2, 4) using probes targeting PAR1 (1, 2) and the duplicated region (3, 4). Note the presence of PAR1 on X and Y chromosomes in the carrier father and control, the PAR flanking probe on the X chromosome in both individuals, but the X insertion signal is found only on the Y chromosome of the carrier father. (D) The heterozygous SNP profile from Illumina sequencing of patient P1 for hg19 chrX:2,680,000-2,830,000. The dashed vertical gray line indicates the pseudoautosomal boundary, the yellow vertical lines illustrate LTR6B positions, gray diamonds illustrate heterozygote SNPs, and the black horizontal lines indicate mean frequencies of all depicted SNPs in the span of the line. Across the top is illustrated chromosome X, with unique X sequence in red, PAR1 reference sequence in purple, and LTR6B's in yellow. Note the presence of heterozygote SNPs in the X specific region, and the SNPs featuring frequencies of 0.33 in the proximal PAR1.

To identify pathogenic copy number variants in patients with developmental disorders, we screened ∼4300 individuals (∼60% male) by microarrays. This screening identified 15 male patients of mainly Belgian origin carrying a duplication with a minimum size of 98,630 bp and of maximum 136,609 bp on Xp22.33 (Figure 1 A). To determine whether the duplication occurred *de novo* or was inherited, arrays were performed on both parents in all 6 families for which parental blood samples could be obtained. We had assumed that the duplicon would have arisen *de novo* or would be inherited from the mother, since males inherit their X chromosome from the mother. However, the duplication was paternally inherited in all families.

Based on the paternal inheritance, we hypothesized the duplicon resided on the Y chromosome. To test this hypothesis and to determine the location of the duplicon, we performed FISH on metaphases from one index, his father, and a male control using probes to target PAR1 and the duplicated region. We observed PAR1 signals on both X and Y chromosomes in all samples (Figure 1 C). The duplicated region was found on the X chromosome in the index, father, and control, but was also found on the Y chromosome of the index and father (Figure 1 C). The FISH analysis demonstrates that the X duplicon is actually located on the Y chromosome at or near the short arm pseudoautosomal region.

Since the duplicated region appeared to be adjacent to the X-linked PAR1, we reasoned that the Y chromosome copy would also be adjacent to the Y-linked PAR1. If so, the regular Y-PAR1 boundary would be disrupted in carriers and a PCR spanning the Y-PAR1 boundary should result in an amplification product in controls, but not in duplication carriers (Figure S1). As expected, the Y-PAR1 boundary specific PCR resulted in an amplification product in normal males, but not in females. In contrast to our hypothesis, the same amplicon was observed in carrier males (Figure S1.). Hence, the duplicon is not a mere extension of Y-PAR1.

To elucidate the exact location of the duplicon on the Y chromosome we performed targeted capture using a BAC spanning the duplicon as a bait, followed by Illumina sequencing for patient P1. The capture resulted in a 1228.4 fold enrichment of the insert region and generated on average 348,529 reads over the bait. Since the BAC spans the duplicon, it was expected that some paired-ends would map back to different locations in the Y chromosome reference sequence, and that some reads would feature split sequences. Unexpectedly, no chimeric pairs or split reads could be detected.

**Figure 2:**
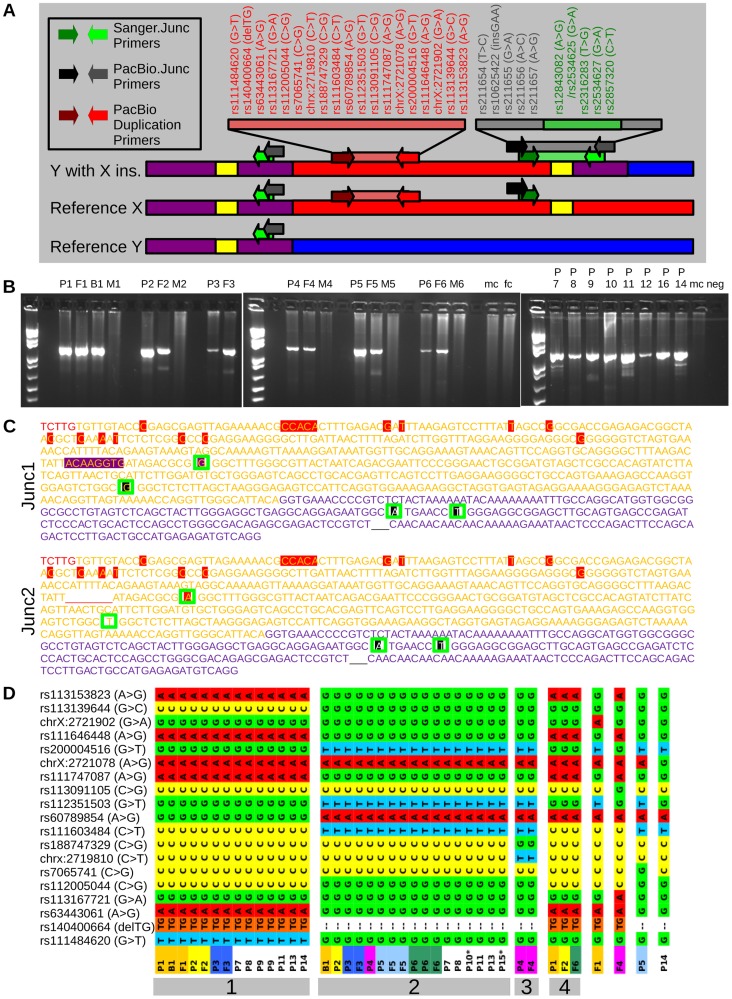
Sequencing to validate the insertion and demonstrate recurrence. (A) Illustrations of a reference Y chromosome, a reference X chromosome, and a Y chromosome with an X insertion. X specific sequence is indicated in red, Y specific sequence in blue, PAR1 reference sequence in purple, and LTR6B's in yellow. Arrows indicate primer pairs, with a bar representing an amplifiable product. The position of the SNPs of this study is shown in the order found in the amplicon. (B) PCRs using the Sanger.Junc primers shows bands for patients (P) and fathers (F), but not mothers (M), male controls (mc), female control (fc) or negative controls (neg), confirming the presence of an X specific insertional translocation in Y. (C) Sequenced amplicons of PCRs from part B, excluding reference upstream/downstream sequence. Red letters are from the X specific reference sequence. Yellow letters are from LTR6B reference sequence with red highlights indicating X specific LTR6B sequence and purple highlights indicating sequence specific for pseudoautosomal LTR6B. Purple letters indicate pseudoautosomal reference sequence. The gap underlined in red indicate bases missing from the X specific LTR6B. In black are annotated SNPs/Indels. In order from the beginning to the end of sequences, green boxes indicate SNP positions for rs2534625/rs12843082, rs2316283, rs2534627, and rs2857320. This Sanger sequencing identified two junction types, indicated as Junc1 and Junc2. (D) Phased haplotypes found through PacBio amplicon sequencing of the PacBio Duplication amplicons, with haplotypes assigned numbers indicated by gray boxes. Families in which both the patient and father were sequenced are color coded. No color indicates a sample in which the father was not sequenced. * Each individual has two haplotypes in the figure, except patients 10 and 15, which had a second unillustrated haplotype with many more variants that more closely resembled Y chromosome sequence.

Upon closer scrutiny of the aligned reads, the PAB region featured three different types of reads: reference Y-PAB reads, reference X-PAB reads, and SNP containing X-PAB reads. The PAB also showed three stretches of heterozygous SNPs: a 33% allele frequency region flanked by two 50% allele frequency regions (Figure 1 D). Since males should have no heterozygous SNPs in their hemizygous X-specific sequences and pseudoautosomal SNPs should show allele ratios of 50% we hypothesized that one portion of the pseudoautosomal region was duplicated. Indeed, the 33% allele frequency region and the proximal 50% allele frequency region represent a duplication event while the distal 50% allele frequency region represents the normal pseudoautosomal SNPs. The breakpoint was delineated by selecting the most proximal (chrX:2,694,303) SNP with an allele frequency of 50% and the most distal (chrX:2,694,429) SNP with an allele frequency of 30%. Interestingly, those SNPs both lie in a long terminal repeat, LTR6B, chrX:2,694,151-2,694,702 (551bp). The most proximal 50% allele frequency SNP was also near a second LTR6B repeat, chrX:2,808,549-2,809,097 (548 bp). These repeats explain that more than 99% of the reads mapping around the SNP frequency changes feature a mapping quality of 0, an indication of reads that map to multiple locations. The presence of these repeats at both sides of the duplicon also explain the absence of chimeric pairs and split reads. The duplicon thus comprises 110 kb of X-specific sequences as well as 5 kb proximal PAR1, resulting in the construct illustrated in Figure 2 A. The 5 kb sequence is present as three copies in the patient (once on his normal X chromosome and twice on his Y chromosome with the X insertion), which explains the 33% SNP ratio profile as well as the presence of three different kinds of reads at the PAB.

To verify the boundary was at the LTR6B in the index patients and their fathers, PCR was performed using one primer located in the distal PAR1 and another located in the X-specific region distal to the duplicon boundary and predicted to span the LTR6B (Figure 2 A, green primers). Not only are these primers separated by 115 kb in the reference genome, but they also have opposite orientations. As expected, the PCR generated an amplicon in carrier males, but not in male or female controls (Figure 2 B). To confirm the presence of the LTR6B in the amplicon, the PCR products were Sanger sequenced, confirming they contained respectively a PAR1 specific fragment, LTR6B, and an X specific sequence (Figure 2 C). These results demonstrate that the duplicon is an insertional translocation from X to Y that is flanked by LTR6B.

### A Reciprocal Deletion

**Figure 3:**
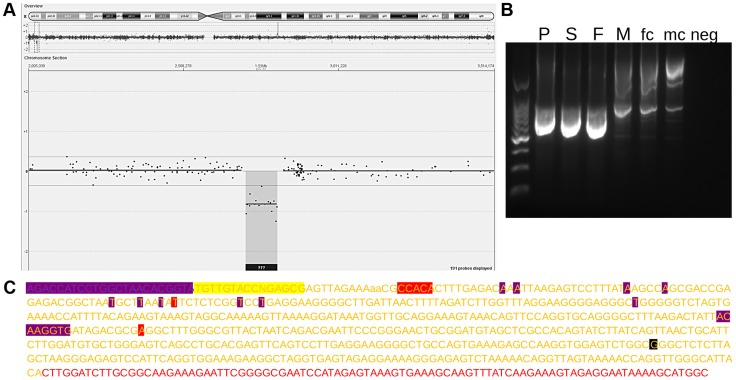
Reciprocal deletion. (A) 180K Custom Microarray aCGH results with the upper track displaying an overview of the log2-ratio of the fluorescent signal across the entire X chromosome and the central track displaying a zoomed in portion of the X chromosome, with the log2-ratio of the fluorescent signals on the Y Axis. (B) PCR bands across the deletion region for the deletion carrying patient (P), father (F), mother (M), sister (S), female control (fc), male control (mc), and negative control (neg). (C) Sequencing of the amplicon in part B for the patient. Red letters indicate X specific reference sequence, yellow letters indicate LTR6B reference sequence, yellow letters highlighted in red indicate sequence specific for X specific LTR6B, yellow letters highlighted in purple indicate sequence specific for pseudoautosomal LTR6B, purple letters highlighted in purple indicate sequence originated from PAR1 that was not sequenced but contains the forward primer site, and yellow letters highlighted in yellow indicate LTR6B sequence that was not sequenced but contains the forward primer site. Highlighted in black are annotated SNPs.

During the initial screening for pathogenic copy number variants we also identified a patient carrying a reciprocal deletion (Figure 3 A). Familial analysis showed both the female index as well as the father and sister to be carriers. We hypothesized the deletion to be reciprocal to the duplication and to have occurred by non-allelic homologous recombination (NAHR) between the LTR6Bs, thus comprising chrX:2694151-2808549 (i.e. from one to the other LTR6B). To confirm this hypothesis we designed a PCR using one primer distal and one proximal of this range. The primer sites are 115 kb apart so the PCR should fail in any individual except those featuring a deletion between them. As expected, the PCR generated an amplicon in the three carriers, but not in male or female controls (Figure 3 B). Therefore, we concluded that the deletion corresponds to the duplication described above. This was verified by Sanger sequencing the index carriers amplicon. The sequence showed an LTR6B featuring an X-specific profile at the region-specific LTR6B positions 2, 10, and 15, and a PAR1-specific profile at positions 3 to 9 and 11 to 14. Position 1 was not sequenced (Figure 3 C). A G was sequenced at rs2534626/2316283. We considered this confirmation that a deletion of chrX:2694151-2808549 results in a merged LTR6B.

### The Insertional Translocation Occurs Recurrently

**Figure 4:**
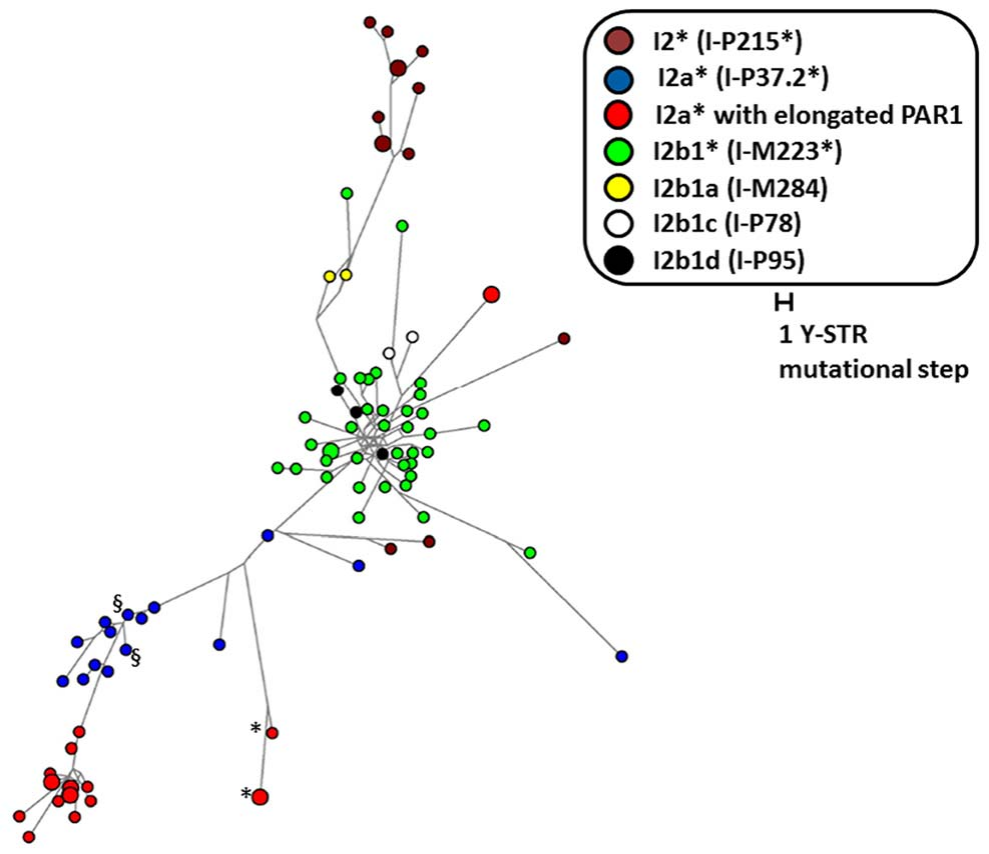
Y Chromosome relatedness of all I2 Y chromosomes. Median joining network based on 26 single-copy Y-STR loci of all I-P215 Y chromosomes observed in the Belgian male population by Larmuseau et al. [53], [54], [56], next to the I2a* (I-P37.2) samples with two PAR-regions. The sizes of the circles are proportional to the haplotype frequency (i.e. 1 or 2 individuals). All I2a* circles with an expanded PAR representing two individuals are father-son pairs. The color of the circles represents the sub-haplogroup to which the haplotype belongs based on Y-SNP typing. * indicates samples identified to contain Junc2 (see Figure 2 C) and § indicates additional samples with an elongated PAR.

The Y-PAR1 extension observed in the different families could have arisen as a single ancient insertional translocation event or might have occurred recurrently. The finding of a reciprocal deletion supports the latter, but to provide additional evidence the relatedness of the Y chromosomes containing the Y-PAR1 extension was determined by chromosome Y SNP/STR typing. All six father Y haplotypes were identical to their index sons. The carriers featured two main Y-chromosomal haplogroups: all 20 carriers of Belgian origin featured haplogroup I (sub-haplogroup I2a*; I-P37.2*) and the two French carriers featured haplogroup R (sub-haplogroup R1b1b2a1a2*; R-P312*)(Figure S2). A comparative Y-STR network analysis with other autochthonously Belgian I2 (I-P215) samples revealed that all but one of the I2a* haplotypes displaying an elongated PAR1 belonged to two closely related clusters (Figure 4). While these two clusters were related to the other I2a* samples earlier observed in the Belgian population the highly distinct I2a*-haplotype appeared to be more closely related to haplogroup I2b1* (I-M223*). Within each of the two clusters of I2a* haplotypes a high paternal kinship was observed as most of their haplotypes differed from each other in less than eight Y-STR loci. According to the mutation rates measured by Ballantyne et al. [23] and the formulae of Walsh [24] the latest common patrilineal ancestors of the largest cluster lived between 7 and 33 generations ago (95% credibility interval), i.e. between 1185 and 1835 (generation span of 25 years) or 855 and 1765 (generation span of 35 years). To determine whether more individuals with the Y-chromosomal sub-haplogroup I2a* carry the described duplicon, we analyzed two additional sub-haplogroup I2a* Belgian individuals (identified as § in Figure 4). Interestingly the Y-extended PAR1 boundary PCR described above also generated amplicons in these individuals. We therefore conclude that the majority of – if not all – I2a* Y chromosomes carry the duplicon.

### The Insertional Translocation in Y Recombines with X

Formal proof that the extended PAR sequence is truly pseudoautosomal would be the demonstration of recombination events within the duplicon between X and Y. We show two lines of circumstantial evidence that this is the case.

First, we amplified ∼5 kb of the distal duplicated region and sequenced the amplicons using long-read single molecule sequencing by PacBio. Haplotypes were phased and all family members were found to share one of three X specific haplotypes (Figure 2 D). All fathers and sons shared at least one similar X specific haplotype, confirming the paternally inherited haplotype. Notably, only family 4 did not have paternal inheritance from the large haplotype groups 1 or 2. This family was also the only family with haplogroup R from Y-Chromosomal STR typing, and was the only non-Belgian family, confirming its distant and independent origin. Oddly, two samples (P10 and P15), did not have a single unphased contig (indicating two identical amplicons) or a single contig with phasing (indicating the same target amplicon sequence with small nucleotide differences). They instead featured one unphased contig resembling the targeted X region (P10 56xcoverage, P15 81xcoverage) and one contig with less coverage (P10 38xcoverage, P15 32xcoverage) matching a highly similar (95%) region of the Y chromosome.

Despite the observation that all I2a* sub-haplogroup members are derived from an ancient NAHR recombination event, there are at least two different extended PAR haplotypes on the Y chromosomes (Table 1). The different haplotypes on Y could have developed through historical mutational events or as a product of recombination between the X chromosome and the duplication insertion on the Y chromosome. Since the two main haplotypes differ by twelve variants we believe recombination is a more likely event.

**Table 1:** Sample overview.

Family	Sample	Sanger.Junc	PacBio.Junc	Duplication.Haplotype	Y-chr haplogroup
1	P1	1	1	1*/4	I
1	F1	1	1	1*/o	I
1	B1	1	1	1*/2	I
2	P2	1	1	1*/2	I
2	F2	1	1	1*/4	I
3	P3	1	1	1/2	I
3	F3		1	1/2	I
4	P4	1	1	3*/2	R
4	F4	1	1	3*/o	R
5	P5	2	2	2*/o	I
5	F5	2	2	2*/2	I
6	P6	1	1	2*/2	I
6	F6	1	1	2*/4	I
7	P7	1	1	1/2	I
8	P8	1	1	1/2	I
9	P9		1	1/1	I
10	P10		2	2/o	I
11	P11		1	1/2	I
12	P12		1c	failed	I
13	P13		1	1/2	I
14	P14	1	1	1/o	I
15	P15		1	2/o	I

If appropriate, each sample has indicated family and relationship: P -patient, B -brother, F -father, Sanger.Junc is the sequencing results of Figure 2 C. PacBio.Junc indicates the breakpoint haplotype deduced from the PacBio amplicons. 1c is the same as junction 1, with the addition of SNP rs211656. Duplication.Haplotype indicates the PacBio phased alleles from the duplicated region found in Figure 2 D (o  =  other haplotype). * indicates the deduced allele of paternal origin in father-son-(brother) pairings. Y-chr haplogroup is the main haplotype groups from chromosome Y SNP-based analyses (Figure S2).

Second, ∼5 kb amplicons spanning the Y-extended PAR junction were PacBio sequenced and the smaller PCR amplicons spanning the boundary were Sanger sequenced. Within the Sanger sequenced region, the reference LTR6B sequences marking the duplicon's borders differ between each other at 15 positions (Figure 2 C). All carrier Sanger sequences had the same distal 13 differences that match the X-specific LTR6B. However, the proximal two differences (SNP rs2534625/rs12843082 and a single indel) were only found in the LTR6B sequence of a subset of carriers. Carriers with the two variants also had an additional non-reference LTR6B sequence variation (rs2316283). We labeled the additional variant containing sequence as Junc1 and the more X-specific sequence as Junc2. All carriers also showed three variants in the pseudoautosomal reference sequence: an A at rs2534627, a T at rs2857320, and 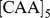 at rs34061732. PacBio amplicon sequencing of the duplicon border identified variants upstream of the Sanger sequencing for rs211654, rs10625422, and rs211655 in all samples with longer amplicon lengths (Table S2, all samples except P10-13). In the region overlapping the Sanger sequences, all PacBio amplicons showed perfect concordance with the Sanger calls. Additionally, a Junc1 carrying an additional SNP (rs211656) was found in a single PacBio amplicon (termed Junc1c in Table 1). To conclude, within the I2a* sub-haplogroup members, two different Y-extended PAR junctions were identified. Junc1 is a recombination between the distal X specific LTR6B and the proximal PAR specific LTR6B, a consequence of the non-allelic recombination event. In Junc2, the original X specific LTR6B is observed. The most likely explanation is that a recombination event occurred in the manner of gene conversion with the X-linked LTR6B serving as the donor and the Y-linked LTR6B as the acceptor reconstituting the original X specific LTR6B sequence. Another possibility is that a recombination event occurred within this LTR6B between the distal and proximal parts of the fusion LTR6B reconstituting the original X specific LTR6B sequence.

## Discussion

PAR1 is common to most eutherian mammals, with a gene order that has been fairly well-conserved since its addition to the pre-existing sex chromosomes that are shared with marsupials [25]. However, the PAB has shifted over evolutionary time and both the size and gene content of PAR1 differ among mammalian species, implying genes within the ancestral PAR have been differentially subsumed into the non-recombining regions in different mammalian lineages [2]. In general, there is evolutionary pressure to expand the non-recombining region resulting in contraction of the PAR. This attrition is attributed to recombination suppression of sex determining region flanking regions. This PAR attrition is counteracted by the addition of new PAR sequences via translocations. Such terminal translocation patterns have shaped the human PAR1 and PAR2 [25]. Here we demonstrate that an apparent male specific X chromosomal copy variant flanking PAR1 represents a PAR1 length polymorphism. The duplication causes a 110 kb proximal extension of PAR1.

**Figure 5:**
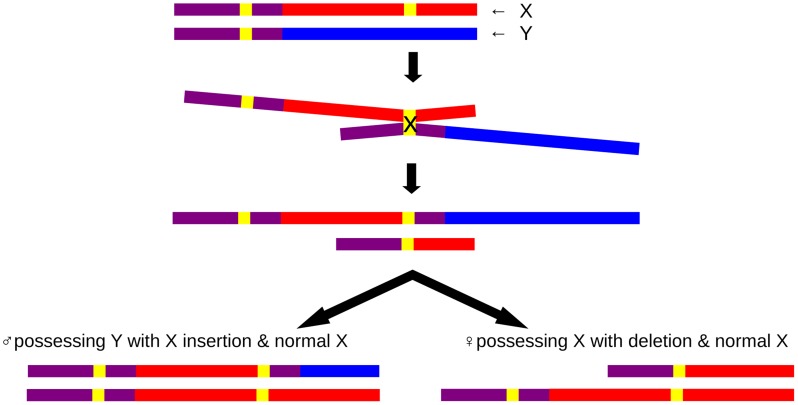
Schematic insertional translocation representation by non-allelic homologous recombination. Purple: PAR1 reference sequence, red: unique X sequence, blue: unique Y sequence; yellow: LTR6B; At the top a paternal set of gonosomes: one normal X and one normal Y chromosome are shown, followed by non-allelic homologous alignment and crossover, resulting in an X chromosome containing a deletion and a Y chromosome containing an X insertion. The bottom illustration demonstrates the situation for male carriers with a Y chromosome containing an X insertion and the reciprocal deletion female carriers with an X chromosome containing a deletion.

The PAR1 extension resulted from an insertional translocation event, caused by non-allelic homologous recombination (NAHR) between the LTR6B repeats, one of which is located within PAR1 and one in the X-specific region (Figure 5). This PAB length polymorphism could reflect either an ancient PAB which has shifted during hominoid evolution or could be a *de novo* event which has occurred during recent human evolution. Several lines of evidence suggest the latter. First, the PAB in great apes and macaques coincides with the human reference PAB. Hence, for this PAB polymorphism to be ancient, it should have arisen during hominoid evolution and subsequently be lost in the majority of our population. Second, the analysis of chromosome Y haplotypes carrying this duplicon shows the presence of this rare variant in different haplogroups which are phylogenetically unrelated (Figure S2) and from different geographic locations, together with an absence of the rare variant in other haplogroups. To be the PAB predecessor, it would have to be lost several times during human evolution. Hence, the most parsimonious explanation is that the PAB polymorphism is caused by independent, but identical, insertional translocation events. In addition, the identification of one family carrying the reciprocal microdeletion which results in a 5 kb reduction of PAR1 on the X chromosome demonstrates that NAHR at the X and Y LTR6B repeats is recurrent.

We not only show sequence homology of the extended PAR region, but provide strong evidence for recombination. This provides formal proof that the extended PAR also acts functionally as a pseudoautosomal sequence. Since all the haplogroup I2a* individuals are ancestrally related, the PAR1 extension is likely to be the consequence of a single insertional translocation event. Nevertheless, different X haplotypes exist in the Y of this haplogroup I2a*. We therefore assume that those haplotypes have been introduced by X-Y recombination. Second, three (four if including Junc1c) of those males have a different junction (Junc2) as compared to the majority of this haplogroup. We deduced that Junc1 is generated by the insertional translocation event and that Junc2 has arisen by X-Y recombination within the X specific LTR6B.

Insertional translocation events are thought to be the consequence of at least three chromosomal breaks resulting in an interchromosomal transposition of a broken fragment (2 breaks) into another chromosome (at least one break). The exact mechanism by which insertional translocations are generated remains, however, to be established. Interestingly, Durkin, et al. [26] showed that several insertional translocation events in cattle genome evolution have occurred via a circular intermediate which subsequently integrated into the receptor chromosome. Here, we present, to our knowledge, the first example of an insertional translocation which is generated by NAHR. Whereas the duplicon is technically an insertional translocation, it has mechanistically arisen because of a terminal translocation event between the long terminal repeat (LTR) in unique X chromosomal sequence and the LTR in the XY homologous region. Several reciprocal balanced and unbalanced translocations have been shown to be the consequence of NAHR between different chromosomes [27]. This translocation event can be considered mechanistically similar. In contrast to the known low copy repeats (LCRs) that generate NAHR events, the LTRs here are extremely short, with only 548 bp homology. Hence, opposite to the general view that only LCRs larger than several kb are drivers of genomic disorders [28], [29], this observation provides further proof that short repeats also have to be considered as drivers of illegitimate recombination [30], [31].

Whether NAHR is a common mechanism for the generation of insertional translocations remains to be determined. Nevertheless, it is tempting to speculate that the proximal PAB expansion detected amongst mouse subspecies also occurred as a consequence of an interstitial NAHR mediated translocation event. The house mouse, *Mus musculus domesticus*, has the smallest PAR amongst those that have been mapped. The PAB is located at about 700 kb from the distal end of the X chromosome, the third intron of the *Mid1* gene and truncates the 5′ end of the Y copy. However, in *Mus musculus castaneus*, a subspecies of the house mouse, the PAB shows a 430 kb shift proximal of the *M.m.domesticus* boundary. The dichotomy in sequence divergence between the proximal and distal segments of the *M.m.castaneus* PAR suggests the proximal segment is a recent translocation of X chromosome sequence to the Y chromosome. Interestingly, the Y specific PAB of *M.m.castaneus* is characterized by a long interspersed nuclear element (LINE1) that is present throughout the mouse genome [32]. Since NAHR between line elements has been suggested as a cause of chromosomal rearrangements [33], [34] it seems plausible that it originated by the same NAHR mechanism. Sequencing of more PABs in other species and populations will probably reveal more pseudoautosomal boundary polymorphisms.

Variation in the PAR boundary is likely to have consequences for the expression of both adjacent genes situated in the duplicon: *XG* and *GYG2* (Figure 1 B). *XG* encodes a surface protein expressed on red blood cells that belongs to a clinically irrelevant blood group system [35]. *GYG2* encodes glycogenin-2, the predominant glycogenin isoform in the liver, which serves as a primer for glycogen synthase [36], [37]. Since the duplication is inherited in all families where the inheritance could be determined and since the duplication can be traced within most likely all I2a* sub-haplogroup members, it seems clear that this variant does not cause developmental anomalies or observable adverse fitness effects. Loss of *XG* and *GYG2* may, however, have biochemical consequences and is likely to result in reduced fitness. Based on the paternal origin of the deletion and the apparent normal phenotype of the father, any effect of nullisomy of both those genes is likely to be minor. However, follow up of this family as well as the detection of more patients with this deletion is required to establish potential phenotypic effects.

In conclusion, we demonstrate that a pseudoautosomal length polymorphism exists in the human population. The extension of the PAR by NAHR presents a novel mechanism shaping sex chromosomal evolution. It seems plausible that such events have occurred frequently during genome evolution. In addition to the already known deceleration of Y chromosome degradation, our results demonstrate a new way of counteracting the processes leading to a loss of Y chromosomes in humans since a proximally extending PAR1 reconstitutes X-borne genetic material thought to be lost from the Y. Thus, if true, current predictions on when the Y chromosome will be lost from humans [38] need to be adapted for the effects of proximally extending PARs. The finding of this length polymorphism also has consequences for statistical genetic analysis in this region since recombination events may alter the haplotype structure periodically.

## Materials and Methods

### Ethics Statement

The study of variants in the human population is part of the analysis of copy number variants coming from an institutional genome wide analysis study. This has been approved by the institutional review board under protocol nr. S55513.

### Sample Collection, Cytogenetic, FISH, and Array Analysis

Blood samples were obtained from 16 patients, 13 parents, and 2 siblings referred for cytogenetic investigation due to the presence of intellectual disability (ID), autism, dysmorphic features or - in one case - subfertility. All families had a geographic origin in Belgium, except family 4 that originated from France. Phenotypes are described in further detail in Table S1. Duplications were confirmed by FISH using BAC clones as previously described [39]. Probe RP11-457M7 was used to target the pseudoautosomal region and probe RP11-146D5 targeted the X-specific duplication.

Samples were analyzed on CytoSure 105K and 180K Custom Microarrays composed of probes from the CytoSure Syndrome Plus v2 array supplemented with probes from the CytoSure ISCA v2 60K array [40], [41]. DNA digestion, labeling, and hybridization were performed according to the manufacturer's protocol.

### PCR and Sanger Sequencing

PCR was used to identify the breakpoints. Primers (Table S2) were designed with Primer3 [42], [43]. Input sequences were masked for interspersed repeat sequences using the RepeatMasker track [44], [45] provided by the UCSC browser [46], [47]. Amplification of fragments was performed using the Platinum Taq DNA Polymerase system (Invitrogen), following the manufacturer's protocol. The thermocycler profile used was: 94°C for 30 sec, followed by 25 cycles at 94°C for 30 sec, 60°C for 30 sec, and 72°C for 2:30 min, with a final extension of 72°C for 1 min.

We performed Sanger sequencing of the breakpoint-spanning amplicons on an ABI 3130xl automated capillary DNA sequencer (Applied Biosystems). First, ExoSAP-IT (USB) treatment was performed according to the manufacturer's protocol. A BigDye Terminator v3.1 Cycle Sequencing Kit (Applied Biosystems) was then used as follows: the sequencing reaction was performed using 2 µl template, 1.5 µl sequencing buffer (5X), 4.5 µl distilled water, 0.5 µl Big Dye, 2.5 µl primer (separate reactions for F and R). Reaction conditions were: 3 min at 96°C followed by 25 cycles at 96°C for 10 sec, 5 sec at 50°C, and 4 min at 60°C. Sequencing products were precipitated using 10 µl sequencing product, 10 µl distilled water, 2 µl NaAcEDTA (1.5 M NaAc + 2.5 mM EDTA) and 80 µl ice cold EtOH (100%). Samples were stored for 15 min at room temperature (RT), and then centrifuged for 30 min at 4°C and 3000 rpm. Supernatant was removed. Samples were centrifuged upside-down for 1 min at 4°C and 1800 rpm. 150 µl ice cold 70% EtOH was added and samples were centrifuged for 10 min at 4°C and 1800 rpm. Supernatant was removed and samples were centrifuged upside-down for 1 min at 4°C and 1800 rpm. Samples were kept for 30 min at RT (dust- and light free). 15 µl of High Dye (formamide) were added before spinning and vortexing samples. Samples were stored for 15 min at RT and denatured for 3 min at 96°C. DNA sequences were visualized using ABI sequence scanner v1.0 (Applied Biosystems).

### BAC Mediated Targeted Paired-End Sequencing

BAC-mediated targeted paired-end sequencing was used to narrow down the breakpoint region. DNA was captured by a BAC mediated pull-down using an adapted protocol of Bashiardes et al. [48]. BAC clone ChrX-32k-3P23 was labeled with BioPrime DNA Labeling System (Invitrogen) according to the manufacturer's protocol. Genomic DNA was sonicated to a fragment size of approximately 350-650 bp and linkers were added. Separately, 300 ng biotin-labeled BAC mixed with 30 µl Cot-1 DNA, 1% 3M Na-Acetate, and 3000 ng fragmented genomic DNA mixed with 1% 3 M Na-Acetate and in 2.5× abs. EtOH were precipitated at −20°C overnight. Samples were centrifuged for 30 min at 4°C and 3000 rpm. Supernatant was removed. Samples were centrifuged upside-down for 1 min at 4°C and 1800 rpm. 150 µl ice cold 70% EtOH were added and samples were centrifuged for 10 min at 4°C and 1800 rpm. Supernatant was removed and samples were centrifuged upside-down for 1 min at 4°C and 1800 rpm. Pellets were dried at 37°C for a few minutes and resuspended in 25 µl nuclease–free water at 37°C for at least 30 min. Samples were transferred to 0.2 ml tubes and denatured and hybridized in a thermocycler as follows: BAC DNA was denatured for 5 min at 95°C and incubated for 15 min at 65°C. 24 µl of 2× hybridization buffer (1.5 M NaCl, 40 mM Na-phosphate buffer pH 7.2, 10 mM EDTA pH8.0, 10× Denhardt's Solution, 0.2% SDS) were added in the cycler and samples were incubated for another hour at 65°C. Genomic DNA was denatured after 40 minutes in another thermocycler for 5 min at 95°C and incubated for 15 min at 65°C. 25 µl of 2× hybridization buffer were added in the cycler. Finally both samples were mixed in the thermocycler by pipetting and hybridized for another 70 h at 65°C. 100 µl magnetic beads (Dynamed M-280 Streptavidin; Invitrogen) were washed twice with 1 ml binding buffer (10 mM Tris-HCl pH 7.5, 1 mM EDTA ph 8.0, 1 M NaCl) and resuspended in 150 µl binding buffer. Hybridization solution was pipetted from cycler to the prepared beads. Tubes were sealed and spun for 30 min upside-down at RT. Beads were pulled down with a magnet, the supernatant removed, and beads were washed once for 15 min in 1 ml 1× SSC, 0.1% SDS on a vibrating table at RT and twice for 15 min in 1 ml 0.1 × SSC 0.1% SDS at 65°C in a vibrating heating block. 50 µl 0.1 M NaOH were added to the beads and they were shook gently for 10 min at RT. Finally the supernatant was pipetted to 1 M Tris-HCL pH 7.5 and the resulting volume of 100 µl was distributed on QIAquick Spin Columns and purified according to the manufacturer's instructions.

Samples were sequenced on a HiSeq 2000 (Illumina) for 2×100 bp reads using the SBS sequencing kit v3 following the manufacturer's protocol. The standard Illumina primary data analysis work-flow was followed for base calling and quality scoring.

Illumina reads were aligned to the human genome (hg19) with BWA v0.5.9 [49] with default settings except that nucleotides with quality score lower than 15 were soft clipped. Read duplicates were discarded after mapping with PICARD MarkDuplicates v1.38 (http://picard.sourceforge.net). Local realignment around indels was performed with RealignerTargetCreator and IndelRealigner from GATK v1.0.4974 [50]–[52]. Finally base quality scores were recalibrated with CountCovariates and Table Recalibration from GATK. The variant frequency of each position of chromosome X between bases 2,680,000 and 2,830,000 was assessed with SNIFER (E. Souche, personal communication). Reads not mapped in proper pair, reads mapped with a mapping quality lower than 30, and nucleotides with quality lower than 20 were discarded. A call was considered heterozygous if its read depth was of at least 100 and the variant frequency was between 25% and 75%.

### Y-chr Haplogroup and Y-STR Typing

In total, 42 Y-STR loci were genotyped for all samples as described in previous studies [53], [54]. However, instead of PowerPlex Y the recent developed PowerPlex Y23 System (Promega Corporation, Madison, WI, USA) was used. All 42-Y-STR haplotypes were submitted to Whit Atheys Haplogroup Predictor [55] to obtain probabilities for the inferred haplogroups. Based on these results, the samples were assigned to specific Y-SNP assays according to previously published protocols [53], [56] to confirm the main haplogroup and to assign the sub-haplogroup to the most accurate level of the latest published Y-chromosomal tree [57], [58].

GenAlEx version 6.5 [59], [60] was used to calculate the differences between all observed haplotypes based on the 42 Y-STR loci. Next, the median joining haplotype network for all the samples belonging to haplogroup I2a* (I-P37.2*) was constructed based on 26 single-copy Y-STRs by NETWORK version 4.5.1.0 [61] (http://www.fluxus-engineering.com) together with all I2 (I-P215) samples already observed in the autochthonous Belgian population by Larmuseau et al. [53], [54], [56]. The network analysis used the weighting scheme described by Qamar et al. [62] due to different mutation rates among the markers based on Ballantyne et al. [23].

### Pacific Biosciences Sequencing and Analyses

Primers (Table S2) were designed with Primer3 to cover chrX:2,718,644-2,723,016 and a combination of chrX:2805180-2809097 plus chrY:2644703-2645415. PacBio specific barcodes with padding sequence were added to the 5′ end of the primers. PCRs were performed using the TaKaRa long range PCR kit by ClonTech. Products were checked on agarose gels, individually purified on MinElute columns (Qiagen), quantified with the Quant-iT PicoGreen dsDNA Assay Kit (Life Technologies), and equimolar amounts pooled. This pool was purified on Qiagen MinElute columns, concentrated, and fragmentation checked on a DNA 12000 chip analyzed on a Bioanalyzer 2100 (Agilent). ∼2 µlg of the pool was prepared for sequencing according to Pacific Biosciences 5 kb protocol using PacBio's DNA Template Prep Kit 2.0 (3 kb-10 kb). The library was first sequenced on a PacBio RS using a DNA/Polymerase Binding Kit 2.0 on a single SMRT cell for a 120 minute movie. The library was sequenced a second time on a PacBio RSII using a DNA/Polymerase Binding Kit P4 on a single SMRT cell for a 180 minute movie. Both runs used PacBio DNA Sequencing Kit 2.0 sequencing reagents. Both SMRTcells were analyzed together using SMRT portal version 2.2's RS_Long_Amplicon_Analysis.1 pipeline with the following non-default settings: minimum sub-read length of 4000, demultiplexing with paired barcodes, and higher stringency on the barcode filtering (30). The assembled amplicon contigs were evaluated by command line BLAST [63] against targeted sequences flanked by 100 N's. Contigs were selected with alignment lengths near to the full target length and a minimum coverage of 30. These contigs were visualized and aligned with ClustalX 2.1 [64] to call variants, ignoring Poly-N length variants of ≥5 nucleotides.

## Supporting Information

Figure S1 Primer design for Y PAR1 extension. Illustrated is a reference X chromosome (A), reference Y chromosome (B), and hypothetical archaic Y chromosome (C). These chromosomes are color coded as: PAR1 sequence in purple, unique X sequence in red, and unique Y sequence in blue. Primers are illustrated as green arrows, with a connecting lighter green box when a PCR product is possible. PCR products (D) are shown for patients (P), male controls (mc), female controls (fc), and a negative control (neg).
(PDF)

Figure S2 The main backbone of the latest published Y chromosomal phylogenetic tree [58]. The nomenclature of all main haplogroups is based on the terminal mutation that defined them. The arrows reveal the haplogroups of Y chromosomes in which extended PAR-regions were observed.
(TIF)

Table S1 Clinical phenotypes.
(PDF)

Table S2 Primers used in the study.
(PDF)

## References

[1] Bachtrog D (2013) Y-chromosome evolution: emerging insights into processes of Y-chromosome degeneration. Nat Rev Genet 14: 113–124. 23329112 10.1038/nrg3366 PMC4120474

[2] Otto SP, Pannell JR, Peichel CL, Ashman TL, Charlesworth D, et al (2011) About PAR: the distinct evolutionary dynamics of the pseudoautosomal region. Trends Genet 27: 358–367. 21962971 10.1016/j.tig.2011.05.001

[3] Lahn BT, Page DC (1999) Four evolutionary strata on the human X chromosome. Science 286: 964–967. 10542153 10.1126/science.286.5441.964

[4] Pandey RS, Wilson Sayres MA, Azad RK (2013) Detecting evolutionary strata on the human X chromosome in the absence of gametologous Y-linked sequences. Genome Biol Evol 5: 1863–1871. 24036954 10.1093/gbe/evt139 PMC3814197

[5] Marais G, Galtier N (2003) Sex chromosomes: how X-Y recombination stops. Curr Biol 13: R641–643. 12932341 10.1016/s0960-9822(03)00570-0

[6] Katsura Y, Iwase M, Satta Y (2012) Evolution of genomic structures on Mammalian sex chromosomes. Curr Genomics 13: 115–123. 23024603 10.2174/138920212799860625 PMC3308322

[7] Aitken RJ, Marshall Graves JA (2002) The future of sex. Nature 415: 963. 11875544 10.1038/415963a

[8] Marshall Graves JA (2006) Sex chromosome specialization and degeneration in mammals. Cell 124: 901–914. 16530039 10.1016/j.cell.2006.02.024

[9] Hughes JF, Skaletsky H, Brown LG, Pyntikova T, Graves T, et al (2012) Strict evolutionary conservation followed rapid gene loss on human and rhesus Y chromosomes. Nature 483: 82–86. 22367542 10.1038/nature10843 PMC3292678

[10] Waters PD, Duffy B, Frost CJ, Delbridge ML, Graves JAM (2001) The human Y chromosome derives largely from a single autosomal region added to the sex chromosomes 80-130 million years ago. Cytogenet Cell Genet 92: 74–79. 11306800 10.1159/000056872

[11] Vermeesch JR, Petit P, Kermouni A, Renauld JC, Van Den Berghe H, et al (1997) The IL-9 receptor gene, located in the Xq/Yq pseudoautosomal region, has an autosomal origin, escapes X inactivation and is expressed from the Y. Hum Mol Genet 6: 1–8. 9002663 10.1093/hmg/6.1.1

[12] Charchar FJ, Svartman M, El-Mogharbel N, Ventura M, Kirby P, et al (2003) Complex events in the evolution of the human pseudoautosomal region 2 (PAR2). Genome Res 13: 281–286. 12566406 10.1101/gr.390503 PMC420362

[13] Flaquer A, Rappold GA, Wienker TF, Fischer C (2008) The human pseudoautosomal regions: a review for genetic epidemiologists. Eur J Hum Genet 16: 771–779. 18398439 10.1038/ejhg.2008.63

[14] Filatov DA, Gerrard DT (2003) High mutation rates in human and ape pseudoautosomal genes. Gene 317: 67–77. 14604793 10.1016/s0378-1119(03)00697-8

[15] Bussell JJ, Pearson NM, Kanda R, Filatov DA, Lahn BT (2006) Human polymorphism and human-chimpanzee divergence in pseudoautosomal region correlate with local recombination rate. Gene 368: 94–100. 16356662 10.1016/j.gene.2005.10.020

[16] Schiebel K, Meder J, Rump A, Rosenthal A, Winkelmann M, et al (2000) Elevated DNA sequence diversity in the genomic region of the phosphatase PPP2R3L gene in the human pseudoautosomal region. Cytogenet Cell Genet 91: 224–230. 11173861 10.1159/000056849

[17] Jorgez CJ, Weedin JW, Sahin A, Tannour-Louet M, Han S, et al (2011) Aberrations in pseudoautosomal regions (PARs) found in infertile men with Y-chromosome microdeletions. J Clin Endocrinol Metab 96: E674–679. 21252244 10.1210/jc.2010-2018 PMC3070254

[18] Ellis NA, Ye TZ, Patton S, German J, Goodfellow PN, et al (1994) Cloning of PBDX, an MIC2-related gene that spans the pseudoautosomal boundary on chromosome Xp. Nat Genet 6: 394–400. 8054981 10.1038/ng0494-394

[19] Ellis NA, Tippett P, Petty A, Reid M, Weller PA, et al (1994) PBDX is the XG blood group gene. Nat Genet 8: 285–290. 7533029 10.1038/ng1194-285

[20] Glaser B, Myrtek D, Rumpler Y, Schiebel K, Hauwy M, et al (1999) Transposition of SRY into the ancestral pseudoautosomal region creates a new pseudoautosomal boundary in a progenitor of simian primates. Hum Mol Genet 8: 2071–2078. 10484777 10.1093/hmg/8.11.2071

[21] Van Laere AS, Coppieters W, Georges M (2008) Characterization of the bovine pseudoautosomal boundary: Documenting the evolutionary history of mammalian sex chromosomes. Genome Res 18: 1884–1895. 18981267 10.1101/gr.082487.108 PMC2593575

[22] Ellis N, Yen P, Neiswanger K, Shapiro LJ, Goodfellow PN (1990) Evolution of the pseudoautosomal boundary in Old World monkeys and great apes. Cell 63: 977–986. 2124175 10.1016/0092-8674(90)90501-5

[23] Ballantyne KN, Goedbloed M, Fang R, Schaap O, Lao O, et al (2010) Mutability of Y-chromosomal microsatellites: rates, characteristics, molecular bases, and forensic implications. Am J Hum Genet 87: 341–353. 20817138 10.1016/j.ajhg.2010.08.006 PMC2933352

[24] Walsh B (2001) Estimating the time to the most recent common ancestor for the Y chromosome or mitochondrial DNA for a pair of individuals. Genetics 158: 897–912. 11404350 10.1093/genetics/158.2.897 PMC1461668

[25] Mangs AH, Morris BJ (2007) The Human Pseudoautosomal Region (PAR): Origin, Function and Future. Curr Genomics 8: 129–136. 18660847 10.2174/138920207780368141 PMC2435358

[26] Durkin K, Coppieters W, Drogemuller C, Ahariz N, Cambisano N, et al (2012) Serial translocation by means of circular intermediates underlies colour sidedness in cattle. Nature 482: 81–84. 22297974 10.1038/nature10757

[27] Ou Z, Stankiewicz P, Xia Z, Breman AM, Dawson B, et al (2011) Observation and prediction of recurrent human translocations mediated by NAHR between nonhomologous chromosomes. Genome Res 21: 33–46. 21205869 10.1101/gr.111609.110 PMC3012924

[28] Liu P, Lacaria M, Zhang F, Withers M, Hastings PJ, et al (2011) Frequency of nonallelic homologous recombination is correlated with length of homology: evidence that ectopic synapsis precedes ectopic crossing-over. Am J Hum Genet 89: 580–588. 21981782 10.1016/j.ajhg.2011.09.009 PMC3188830

[29] Liu P, Carvalho CM, Hastings PJ, Lupski JR (2012) Mechanisms for recurrent and complex human genomic rearrangements. Curr Opin Genet Dev 22: 211–220. 22440479 10.1016/j.gde.2012.02.012 PMC3378805

[30] Shaw CJ, Lupski JR (2005) Non-recurrent 17p11.2 deletions are generated by homologous and non-homologous mechanisms. Hum Genet 116: 1–7. 15526218 10.1007/s00439-004-1204-9

[31] Luo Y, Hermetz KE, Jackson JM, Mulle JG, Dodd A, et al (2011) Diverse mutational mechanisms cause pathogenic subtelomeric rearrangements. Hum Mol Genet 20: 3769–3778. 21729882 10.1093/hmg/ddr293 PMC3168286

[32] White MA, Ikeda A, Payseur BA (2012) A pronounced evolutionary shift of the pseudoautosomal region boundary in house mice. Mamm Genome 23: 454–466. 22763584 10.1007/s00335-012-9403-5 PMC3519421

[33] Koumbaris G, Hatzisevastou-Loukidou H, Alexandrou A, Ioannides M, Christodoulou C, et al (2011) FoSTeS, MMBIR and NAHR at the human proximal Xp region and the mechanisms of human Xq isochromosome formation. Hum Mol Genet 20: 1925–1936. 21349920 10.1093/hmg/ddr074 PMC3428953

[34] Higashimoto K, Maeda T, Okada J, Ohtsuka Y, Sasaki K, et al (2013) Homozygous deletion of DIS3L2 exon 9 due to non-allelic homologous recombination between LINE-1s in a Japanese patient with Perlman syndrome. Eur J Hum Genet 21: 1316–1319. 23486540 10.1038/ejhg.2013.45 PMC3798850

[35] Johnson NC (2011) XG: the forgotten blood group system. Immunohematology 27: 68–71. 22356523

[36] Mu J, Skurat AV, Roach PJ (1997) Glycogenin-2, a novel self-glucosylating protein involved in liver glycogen biosynthesis. J Biol Chem 272: 27589–27597. 9346895 10.1074/jbc.272.44.27589

[37] Mu J, Roach PJ (1998) Characterization of human glycogenin-2, a self-glucosylating initiator of liver glycogen metabolism. J Biol Chem 273: 34850–34856. 9857012 10.1074/jbc.273.52.34850

[38] Marshall Graves JA (2002) The rise and fall of SRY. Trends Genet 18: 259–264. 12047951 10.1016/s0168-9525(02)02666-5

[39] Vermeesch JR, Mertens G, David G, Marynen P (1995) Assignment of the human glypican gene (GPC1) to 2q35-q37 by fluorescence in situ hybridization. Genomics 25: 327–329. 7774946 10.1016/0888-7543(95)80152-c

[40] Srisupundit K, Brady PD, Devriendt K, Fryns JP, Cruz-Martinez R, et al (2010) Targeted array comparative genomic hybridisation (array CGH) identifies genomic imbalances associated with isolated congenital diaphragmatic hernia (CDH). Prenat Diagn 30: 1198–1206. 21064195 10.1002/pd.2651

[41] Vermeesch JR, Melotte C, Froyen G, Van Vooren S, Dutta B, et al (2005) Molecular karyotyping: array CGH quality criteria for constitutional genetic diagnosis. J Histochem Cytochem 53: 413–422. 15750031 10.1369/jhc.4A6436.2005

[42] Untergasser A, Cutcutache I, Koressaar T, Ye J, Faircloth BC, et al (2012) Primer3–new capabilities and interfaces. Nucleic Acids Res 40: e115. 22730293 10.1093/nar/gks596 PMC3424584

[43] Koressaar T, Remm M (2007) Enhancements and modifications of primer design program Primer3. Bioinformatics 23: 1289–1291. 17379693 10.1093/bioinformatics/btm091

[44] Jurka J (2000) Repbase update: a database and an electronic journal of repetitive elements. Trends Genet 16: 418–420. 10973072 10.1016/s0168-9525(00)02093-x

[45] Smit AFA, Hubley R, Green P (1996–2010). RepeatMasker Open-3.0. URL http://www.repeatmasker.org.

[46] Kent WJ, Sugnet CW, Furey TS, Roskin KM, Pringle TH, et al (2002) The human genome browser at UCSC. Genome Res 12: 996–1006. 12045153 10.1101/gr.229102 PMC186604

[47] Karolchik D, Barber GP, Casper J, Clawson H, Cline MS, et al (2014) The UCSC Genome Browser database: 2014 update. Nucleic Acids Res 42: D764–770. 24270787 10.1093/nar/gkt1168 PMC3964947

[48] Bashiardes S, Veile R, Helms C, Mardis ER, Bowcock AM, et al (2005) Direct genomic selection. Nat Methods 2: 63–69. 16152676 10.1038/nmeth0105-63

[49] Li H, Durbin R (2009) Fast and accurate short read alignment with Burrows-Wheeler transform. Bioinformatics 25: 1754–1760. 19451168 10.1093/bioinformatics/btp324 PMC2705234

[50] McKenna A, Hanna M, Banks E, Sivachenko A, Cibulskis K, et al (2010) The Genome Analysis Toolkit: a MapReduce framework for analyzing next-generation DNA sequencing data. Genome Res 20: 1297–1303. 20644199 10.1101/gr.107524.110 PMC2928508

[51] DePristo MA, Banks E, Poplin R, Garimella KV, Maguire JR, et al (2011) A framework for variation discovery and genotyping using next-generation DNA sequencing data. Nat Genet 43: 491–498. 21478889 10.1038/ng.806 PMC3083463

[52] Van der Auwera GA, Carneiro M, Hartl C, Poplin R, del Angel G, et al (2013) From FastQ Data to High-Confidence Variant Calls: The Genome Analysis Toolkit Best Practices Pipeline. Curr Protoc Bioinformatics 43: 11.10.1–11.10.33. 25431634 10.1002/0471250953.bi1110s43 PMC4243306

[53] Larmuseau MH, Vanderheyden N, Jacobs M, Coomans M, Larno L, et al (2011) Micro-geographic distribution of Y-chromosomal variation in the central-western European region Brabant. Forensic Sci Int Genet 5: 95–99. 21036685 10.1016/j.fsigen.2010.08.020

[54] Larmuseau MH, Vanoverbeke J, Gielis G, Vanderheyden N, Larmuseau HF, et al (2012) In the name of the migrant father–analysis of surname origins identifies genetic admixture events undetectable from genealogical records. Heredity (Edinb) 109: 90–95. 22511074 10.1038/hdy.2012.17 PMC3400745

[55] Athey WT (2006) Haplogroup prediction from Y-STR values using a Bayesian-allele-frequency approach. Journal of Genetic Genealog 2: 34–39.

[56] Larmuseau MH, Ottoni C, Raeymaekers JA, Vanderheyden N, Larmuseau HF, et al (2012) Temporal differentiation across a West-European Y-chromosomal cline: genealogy as a tool in human population genetics. Eur J Hum Genet 20: 434–440. 22126748 10.1038/ejhg.2011.218 PMC3306861

[57] Van Geystelen A, Decorte R, Larmuseau MH (2013) AMY-tree: an algorithm to use whole genome SNP calling for Y chromosomal phylogenetic applications. BMC Genomics 14: 101. 23405914 10.1186/1471-2164-14-101 PMC3583733

[58] Van Geystelen A, Decorte R, Larmuseau MH (2013) Updating the Y-chromosomal phylogenetic tree for forensic applications based on whole genome SNPs. Forensic Sci Int Genet 7: 573–580. 23597787 10.1016/j.fsigen.2013.03.010

[59] Peakall R, Smouse PE (2006) GENALEX 6: genetic analysis in Excel. Population genetic software for teaching and research. Molecular Ecology Notes 6: 288–295. 10.1093/bioinformatics/bts460 PMC3463245 22820204

[60] Peakall R, Smouse PE (2012) GenAlEx 6.5: genetic analysis in Excel. Population genetic software for teaching and research–an update. Bioinformatics 28: 2537–2539. 22820204 10.1093/bioinformatics/bts460 PMC3463245

[61] Bandelt HJ, Forster P, Rohl A (1999) Median-joining networks for inferring intraspecific phylogenies. Mol Biol Evol 16: 37–48. 10331250 10.1093/oxfordjournals.molbev.a026036

[62] Qamar R, Ayub Q, Mohyuddin A, Helgason A, Mazhar K, et al (2002) Y-chromosomal DNA variation in Pakistan. Am J Hum Genet 70: 1107–1124. 11898125 10.1086/339929 PMC447589

[63] Altschul SF, Gish W, Miller W, Myers EW, Lipman DJ (1990) Basic local alignment search tool. J Mol Biol 215: 403–410. 2231712 10.1016/S0022-2836(05)80360-2

[64] Larkin MA, Blackshields G, Brown NP, Chenna R, McGettigan PA, et al (2007) Clustal W and Clustal X version 2.0. Bioinformatics 23: 2947–2948. 17846036 10.1093/bioinformatics/btm404

