# Correction: Pseudoautosomal Region 1 Length Polymorphism in the Human Population

**DOI:** 10.1371/journal.pgen.1004889

**Published:** 2014-12-03

**Authors:** The PLOS Genetics Staff

Two errors are included in the published manuscript.

In the abstract the sentence “Subsequent genomic analysis demonstrated that an insertional translocation of X chromosomal sequence into theMa Y chromosome generates an extended PAR.” Should read “Subsequent genomic analysis demonstrated that an insertional translocation of X chromosomal sequence into the Y chromosome generates an extended PAR.”

In the last paragraph of materials and methods, the sentence “∼2 µlg of the pool was prepared for sequencing according to Pacific Biosciences 5 kb protocol using PacBio's DNA Template Prep Kit 2.0 (3 kb-10 kb)” should instead have the value ∼4 µg.
