# Supplementary figures and images for: Pseudoautosomal Region 1 Length Polymorphism in the Human Population
Source: PLoS Genet. 2014 Nov 6;10(11):e1004578. doi: 10.1371/journal.pgen.1004578 (PMC4222609; doi:10.1371/journal.pgen.1004578)

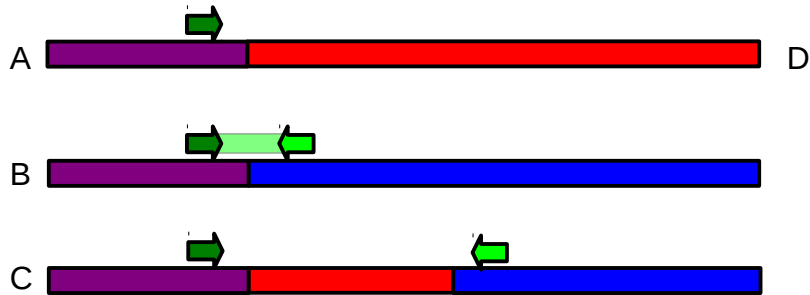

D

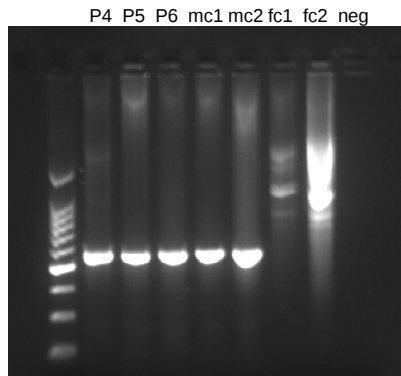

Supplement: Figure S1 — Primer design for Y PAR1 extension. Illustrated is a reference X chromosome (A), reference Y chromosome (B), and hypothetical archaic Y chromosome (C). These chromosomes are color coded as: PAR1 sequence in purple, unique X sequence in red, and unique Y sequence in blue. Primers are illustrated as green arrows, with a connecting lighter green box when a PCR product is possible. PCR products (D) are shown for patients (P), male controls (mc), female controls (fc), and a negative control (neg). (PDF) [file pgen.1004578.s001.pdf]
