# Supplementary material for: Pseudoautosomal Region 1 Length Polymorphism in the Human Population
Source: PLoS Genet. 2014 Nov 6;10(11):e1004578. doi: 10.1371/journal.pgen.1004578 (PMC4222609; doi:10.1371/journal.pgen.1004578)
Supplement: Table S1 — Clinical phenotypes. (PDF) [file pgen.1004578.s003.pdf]

Family	Sample	Clinical description
1	P1	moderate ID, autism, macrocephaly
1	F1	no info
1	M1	no info
1	B1	autism spectrum disorder (ASD), macrocephaly
2	P2	subfertility
2	F2	no info
2	M2	no info
3	P3	ASD
3	F3	no info
4	P4	bilateral coloboma, strabismus, psychomotor instability, speech delay, attention deficit disorder
4	F4	no info
4	M4	no info
5	P5	ID, microcephaly, seizures (15q13.3 microdeletion syndrome)
5	F5	no info
5	M5	school drop-out (carrier of 15q13.3 microdeletion)
6	P6	developmental delay, ASD, arachnoid cyst, macrocephaly
6	F6	no info
6	M6	no info
	P7	ID
	P8	ID and behavioural abnormality (agression)
	P9	ID, ASD
	P10	moderate ID, behavioural abnormality (verbal agression), psychosis, kyphosis
	P11	ID, facial dysmorphism (small palpebral fissures, long philtrum, retrognathia)
	P12	ASD and facial dysmorphism (epicanthal folds, small palpebral fissures, thin upper lip, flat long philtrum, micrognathia)
	P13	developmental delay, ASD, hypotonia, seizures, nystagmus
	P14	developmental delay, failure to thrive, facial dysmorphism
	P15	mild developmental delay, hypotonia, epicanthal folds and short stature
Deletion	P_del	behavioral abnormaility, mild ID, dysmorphism (straight eyebrows, broad neck, low occipital hairline, concave nails) and scoliosis
	F_del	unaffected
	M_del	unaffected
	S_del	unaffected
