# Supplementary material for: Pseudoautosomal Region 1 Length Polymorphism in the Human Population
Source: PLoS Genet. 2014 Nov 6;10(11):e1004578. doi: 10.1371/journal.pgen.1004578 (PMC4222609; doi:10.1371/journal.pgen.1004578)
Supplement: Table S2 — Primers used in the study. (PDF) [file pgen.1004578.s004.pdf]

Primers Used in This Study

Forward primer	Reverse Primer	Target
GGGGTCCCGAGATTTATGTT	TCTCGATCTCCTGACCTCGT	Supplemental Figure 1
TGGCAATGTTACTGGAGACG	TTCTTCATTCTTCATTTTCATCCA	Figure 2 A Green (Sanger Junction)
BARCODE(1-24)+GCCCATCCTCAGAACTCACT	BARCODE(1-24)+AGGCATCCTCGAAAACAAGC	Figure 2 A Red (PacBio duplicated region)
BARCODE(21-24)+ACATGGTAGACGCCTGTTCA	BARCODE(21-24)+TTCTTCATTCTTCATTTTCATCCA	Figure 2A Grey (PacBio Junction -samples P10,P11,P12,P13)
BARCODE(1-20)+AAATGAACGCTAAGCCCCAC	BARCODE(1-20)+TTCTTCATTCTTCATTTTCATCCA	Figure 2A Grey (PacBio Junction -other samples than above row)
AGACCATCCTGGCTAACACG	TCCAACCCCTGTTTAGCATC	Figure 3 B (Reciprocal deletion)

BARCODES are PacBio recommended barcodes 1-24 with padding
